# Effects of Essential Amino Acid Supplementation on Clinical Outcomes in the Conservative Management of Knee Osteoarthritis

**DOI:** 10.7759/cureus.89582

**Published:** 2025-08-07

**Authors:** Atsushi Hoki, Shingo Kurihara, Yoshikazu Matsuda

**Affiliations:** 1 Department of Physical Therapy, Matsuda Orthopedic Clinic, Kumagaya, JPN; 2 Department of Orthopedic Surgery, Matsuda Orthopedic Clinic, Kumagaya, JPN

**Keywords:** conservative treatment, essential amino acid, knee osteoarthritis, nutrients, supplementation

## Abstract

Background: The effect of supplementation of essential amino acids (EAAs) in knee osteoarthritis (OA) remains unclear. This study aimed to evaluate whether supplementation with EAA improves pain, patient-reported outcome measures, gait function, and quadriceps muscle volume in patients undergoing conservative treatment for knee OA.

Methods: A retrospective cohort study was conducted on outpatients undergoing physical therapy from April 2024 to March 2025. Inclusion criteria were patients who started physical therapy for knee OA, with exclusion of patients taking other supplements or having severe medical conditions. Patients who received EAA at the beginning of physical therapy were assigned to the EAA group (8 g/day supplementation), while those who did not were assigned to the control group. The primary outcome was the maximum value on the visual analogue scale (VAS). Secondary outcomes included the Knee injury and Osteoarthritis Outcome Score (KOOS) subscales, gait speed and steps, quadriceps torque, and quadriceps volume measured at baseline and 12 weeks.

Results: Of the 51 patients initially included, 36 patients were ultimately analyzed (EAA group: n = 17, control group: n = 19). No significant differences were observed in VAS, KOOS subscales, and quadriceps torque between groups. However, the EAA group demonstrated significantly greater improvements in 6-meter gait speed, step count, and quadriceps volume compared to the control group. No adverse effects were observed associated with EAA supplementation.

Conclusions: EAA supplementation in the conservative treatment of knee OA demonstrated significant improvements in gait function and quadriceps muscle volume compared to the control group. These results suggest the efficacy of EAA supplementation in the conservative management of knee OA.

## Introduction

Knee osteoarthritis (OA) is becoming more prevalent with increasing age, affecting approximately 13% of women and 10% of men aged 60 years and older globally, creating a significant social burden [1]. Conservative management serves as the primary treatment modality for the majority of knee OA patients [2], with traditional approaches including weight reduction, physical therapy, injections, oral analgesics, and topical medications according to clinical guidelines [3,4]. However, recent studies have revealed beneficial effects of nutritional supplementation, particularly protein, amino acids, and peptides, in the conservative management of knee osteoarthritis [5-9].

Previous studies in outpatient conservative treatment settings have reported beneficial effects of protein powder, collagen-based supplements, and amino acids on both knee function and patient-reported outcomes (PROMs) [8-10]. In the perioperative setting, essential amino acid (EAA) supplementation has also been shown to promote early recovery and preserve muscle volume following knee surgery [5,11-13]. EAAs, which include amino acids such as leucine, isoleucine, and valine, cannot be synthesized by the human body and must be obtained from the diet. They stimulate muscle protein synthesis and have been shown to be the primary driver of muscle anabolism in older adults [13-15]. While EAA supplementation has shown benefits in perioperative recovery after knee surgery, its effects in non-surgical, outpatient conservative treatment of knee OA have not been investigated. Compared to whole-protein supplements, EAA offers several advantages, including faster digestion and absorption, as well as the elimination of concerns related to lactose intolerance, thereby avoiding contraindications associated with dairy protein use [7,14,16]. In addition, it can achieve similar or better efficacy at lower doses, making it suitable for a broader range of patients [16]. Furthermore, reduced dosage requirements may improve patient compliance, warranting validation of its clinical utility [6,17].

This study aimed to evaluate the effect of EAA supplementation in patients undergoing conservative treatment for knee osteoarthritis. The primary objective was to assess changes in patient-reported pain using the visual analogue scale (VAS). Secondary objectives included evaluating changes in functional outcomes such as the Knee injury and Osteoarthritis Outcome Score (KOOS) subscales, gait speed, step count, quadriceps muscle torque, and quadriceps muscle volume. We hypothesized that EAA supplementation would be associated with improvements in pain, physical function, and muscle characteristics compared to standard care alone.

## Materials and methods

Study design

We conducted a retrospective cohort study of outpatients with knee OA who visited Matsuda Orthopedic Clinic between April 1, 2024, and March 31, 2025. Ethical approval was obtained from the Matsuda Orthopedic Clinic Institutional Review Board (approval number 20250705). This study was conducted as part of routine clinical care using anonymized retrospective data, and no study-specific informed consent was required in accordance with the institutional ethics policy. At the initial visit, all patients were informed of available treatment options, including surgical and conservative management. Patients who selected conservative treatment with physical therapy were considered eligible for inclusion. The inclusion criterion was participation in a physical therapy program for knee OA. Exclusion criteria included prior knee arthroplasty on the affected side, use of other supplements, renal dysfunction, uncontrolled hypertension, and neurological or cognitive impairment. Patients in the physical therapy program were offered the option to purchase EAA powder within the clinic. Those who opted for supplementation were included in the EAA group, while those without supplementation were included in the control group. Both groups received the same physical therapy regimen without randomization. The start date of physical therapy was defined as day 0, and patients were followed for up to 90 days. Patients were excluded if they underwent any change in treatment policy (e.g., knee surgery or biologic therapy) during the follow-up period. In total, three patients in the EAA group and six patients in the control group were lost to follow-up, and these patients were excluded from the final analysis.

Conservative treatment

On day 0, baseline outcomes, including VAS, PROMs, grip strength, quadriceps torque, and quadriceps volume, were assessed. The physical therapy program was based on evidence-based clinical practice guidelines issued by the American Academy of Orthopaedic Surgeons for the management of knee OA [18]. The program included supervised sessions focused on lower limb strengthening (e.g., quadriceps setting, straight-leg raises, hip abduction), functional training (e.g., step-ups, standing calf raises), and education on home-based exercises. As this was a retrospective study, analgesic medications were prescribed according to individual patient needs as part of routine clinical care, without protocolized control, and over-the-counter medications were not restricted.

Nutritional supplementation

Participants in the EAA group received oral supplementation with powdered EAA (ESporitamin®; EA Pharma, Tokyo, Japan) at a dose of 8 g/day, administered as 4 g twice daily between meals (once in the morning and once in the afternoon). Each 8 g dose of the supplement contained the following amino acids: threonine (360 mg), lysine (672 mg), isoleucine (536 mg), valine (536 mg), methionine (536 mg), tryptophan (184 mg), phenylalanine (360 mg), leucine (608 mg), histidine (280 mg), arginine (560 mg), and glycine (968 mg). The remaining component was starch (2,400 mg). Although the Japanese government has approved the commercial packaging of this EAA supplement for the prevention of perioperative undernutrition, its use in the present study was not covered by public health insurance, as all participants were outpatients. Therefore, the supplement was purchased at the patients' own expense. At the time of data collection in April 2024, the supplement was priced at approximately 0.17 USD per gram, amounting to around 38.7 USD for a 28-day supply. EAA supplementation was initiated on the same day as the start of physical therapy. The dosage of 8 g/day was chosen for practical reasons. The supplement is available in 2 g units, and the 8 g/day dosage was chosen to ensure simple and manageable intake, aiming to support compliance in outpatient care.

Outcomes

**Figure 1:**
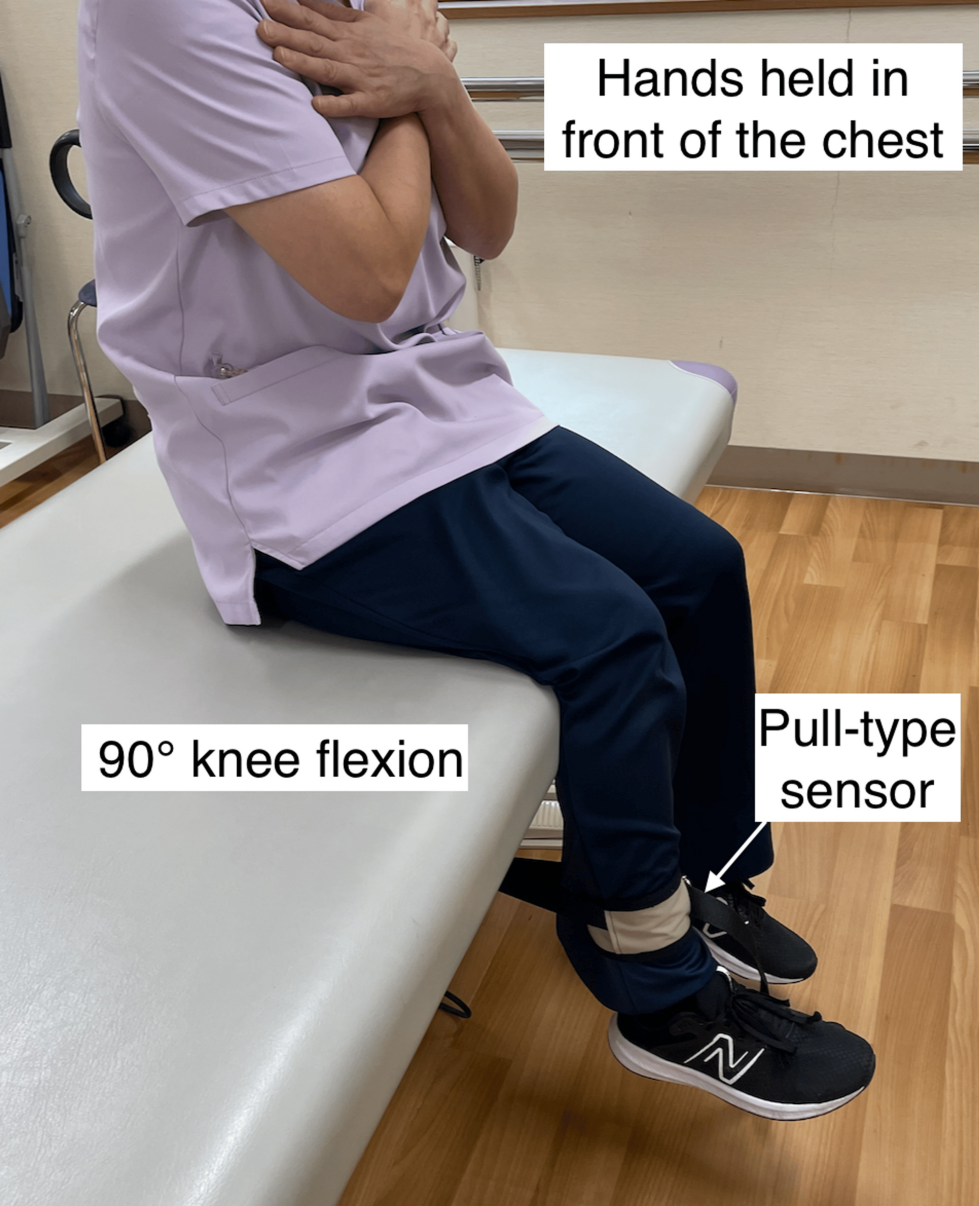
Measurement of quadriceps muscle torque. Patients sat with their knees flexed at 90°, their hands held in front of their chest, and their feet not in contact with any surface. Isometric quadriceps strength was measured using a pull-type hand-held dynamometer. Original image by the authors.

**Figure 2:**
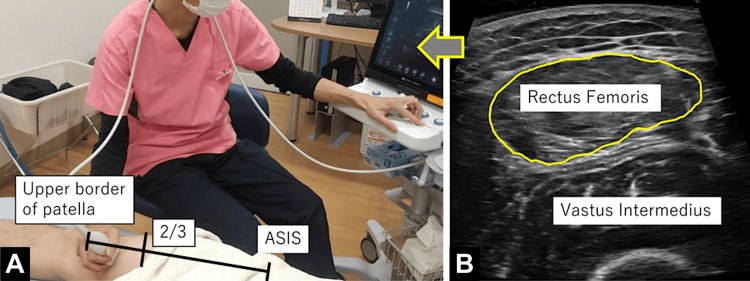
Ultrasound image and examination scene of the rectus femoris muscle. (A) Examination setup. The anatomical measurement point is marked at approximately two-thirds the distance from the anterior superior iliac spine (ASIS) to the upper border of the patella. (B) Ultrasound image. Cross-sectional view of the rectus femoris and vastus intermedius muscles, with the rectus femoris area outlined in yellow. Original image by the authors.

The primary outcome was maximum VAS, defined as the worst pain experienced in the past week and recorded as a self-reported integer from 0 to 10. Secondary outcomes included PROMs using the KOOS subscales: symptoms, pain, activities of daily living (ADL), sport and recreation function (SP), and quality of life (QOL). Additionally, physical activity measures included gait speed and step count during 6-meter walking on level ground. Participants were instructed to walk at their usual comfortable pace. Quadriceps torque was measured using a pull-type hand-held dynamometer (Figure 1). For imaging evaluation, the cross-sectional area of the rectus femoris muscle was measured using ultrasonography, following a previously described method [13]. Measurements were performed with the patient in a relaxed supine position and the knee extended. The scanning site was located at approximately two-thirds of the distance from the anterior superior iliac spine to the superior border of the patella (Figure 2). Three measurements were taken by two physical therapists, and the median value was adopted. These measurements were performed at days 0, 4, 8, and 12 weeks, respectively. These secondary outcomes were selected based on previous studies showing their relevance in evaluating physical function and muscle adaptation with EAA supplementation [5,13].

Statistical analysis

First, we described the baseline characteristics and clinical outcomes of all eligible patients. Baseline variables included age, sex, body mass index (BMI), affected side (left, right, or bilateral), Kellgren-Lawrence (KL) grade, and valgus knee alignment. Grip strength was included as an indicator of overall physical potential at baseline, given its reported association with functional outcomes in patients with knee OA and those undergoing total knee arthroplasty (TKA) [19].

For clinical outcomes, the differences between baseline and 12-week values were calculated for each participant and compared between the EAA and control groups using t-tests. Continuous variables are presented as means with standard deviations, and categorical variables as frequencies and percentages. Between-group comparisons were conducted using t-tests for continuous variables and the chi-square test or Fisher's exact test for categorical variables. A two-sided p-value of <0.05 was considered statistically significant. All analyses were performed using R version 4.3.2 (R Foundation for Statistical Computing, Vienna, Austria).

For the primary analysis, missing outcome data at 12 weeks were imputed using the Last Observation Carried Forward (LOCF) method, with the eight-week value carried forward if the 12-week data were missing but the eight-week data were available. To assess the robustness of our findings, a sensitivity analysis was conducted without imputation.

## Results

Study flow

**Figure 3:**
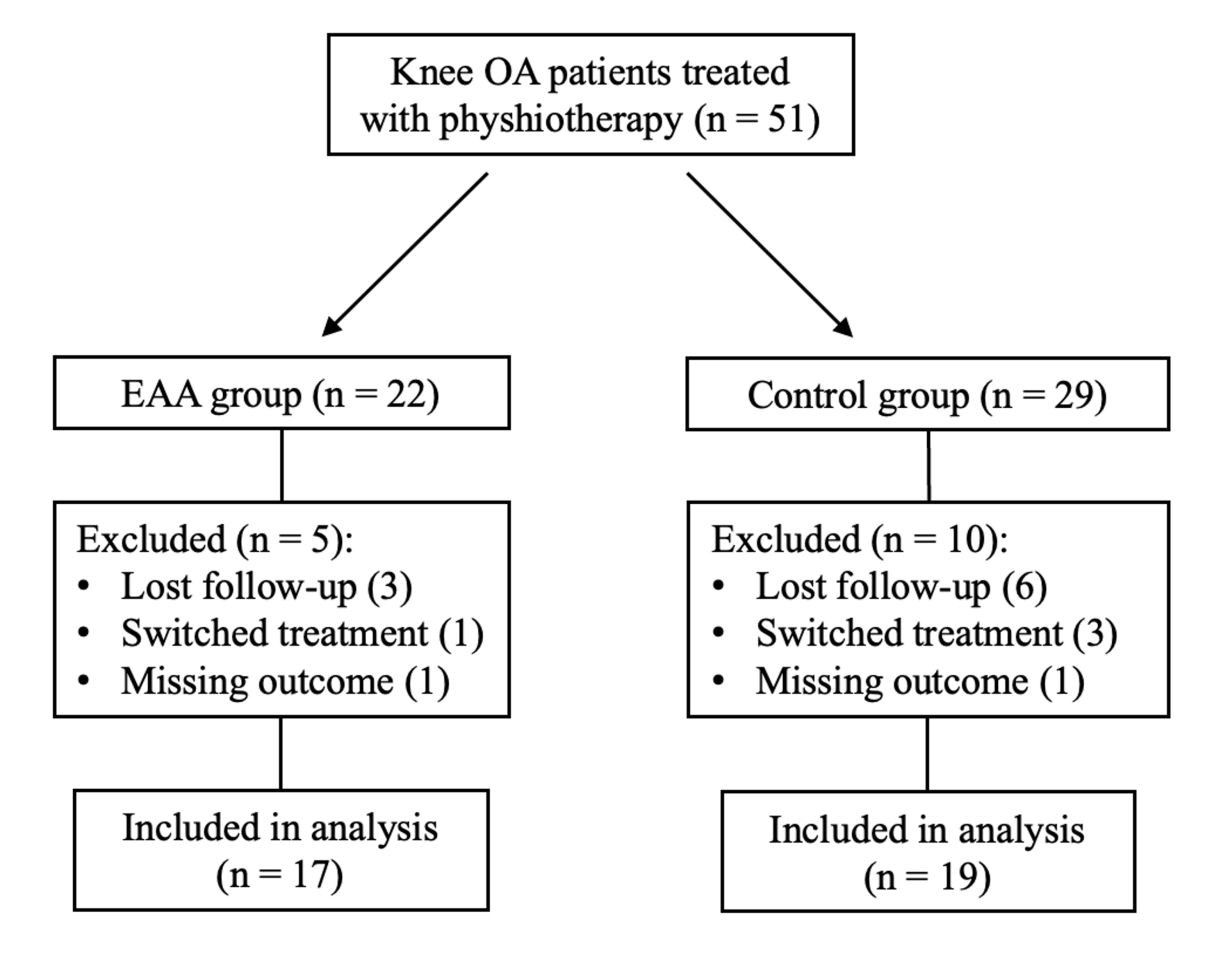
Flowchart of the study. OA: osteoarthritis; EAA: essential amino acid

During the study period, 51 patients who underwent physical therapy with a diagnosis of OA were divided into two groups: 22 patients were assigned to the EAA group, and 29 patients were assigned to the control group. After 12 weeks, 17 in the EAA and 19 in the control group were finally analyzed (Figure 3).

Baseline characteristics

The mean age of the patients was 74.5 years, and 69.4% were women. The baseline characteristics showed no significant difference between the two groups (Table 1).

**Table 1:** Baseline characteristics of the study participants. Data are presented as mean ± standard deviation (SD) or n (%). P-values represent comparisons between groups, with p < 0.05 considered statistically significant. Note: The KL grade was defined based on the higher grade between the right and left knees. EAA, essential amino acid; SD, standard deviation; KL, Kellgren–Lawrence

Variables	EAA (n = 17)	Control (n = 19)	Total (n = 36)	P-value	Test statistic
Age (years), mean (SD)	74.9 (3.9)	74.2 (6.1)	74.5 (5.2)	0.68	t = -0.43
Female, n (%)	13 (76.5)	12 (63.2)	25 (69.4)	0.62	χ² = 0.25
Body mass index, mean (SD)	24.9 (5.4)	25.1 (3.8)	25.5 (4.1)	0.91	t = 0.12
Affected side, n (%)					
Left	5 (29.4)	5 (26.3)	10 (27.8)	0.96	χ² = 0.08
Right	3 (17.6)	4 (21.1)	7 (19.4)		
Bilateral	9 (52.9)	10 (52.6)	19 (52.8)		
KL grade, n (%)					
2	3 (15.8)	1 (5.9)	4 (11.1)	0.51	χ² = 3.31
3	9 (47.4)	7 (41.2)	16 (44.4)		
4	7 (36.8)	9 (52.9)	16 (44.4)		
Valgus, n (%)	3 (17.6)	2 (10.5)	5 (13.9)	0.56	χ² = 0.36
Grip (kg), mean (SD)					
Left	26.4 (6.9)	26.0 (8.7)	28.4 (7.6)	0.89	t = -0.14
Right	28.1 (7.3)	28.7 (8.1)	26.2 (7.8)	0.79	t = 0.27

Outcomes

The longitudinal changes in each group showed that in the EAA group, significant improvements were observed in the KOOS SP and QOL subscales, while the control group demonstrated significant improvements in the KOOS Symptom and Pain subscales. Other KOOS domains also tended to improve in both groups. In addition, gait speed, step count, and quadriceps muscle volume increased significantly only in the EAA group (Table 2).

**Table 2:** Changes in clinical outcomes within each group from baseline to 12 weeks. Data are presented as mean ± standard deviation (SD). P-values were calculated using paired Student's t-tests to compare baseline and 12-week values within each group. Corresponding t-values are described. P-values < 0.05 were considered statistically significant. EAA, essential amino acid; VAS, visual analogue scale; SD, standard deviation; KOOS, Knee injury and Osteoarthritis Outcome Score; ADL, activities of daily living; SP, sports and recreational activities; QOL, quality of life; cm², square centimeters.

Variable	EAA group (N = 17)				Control group (N = 19)			
	Baseline	12 weeks	P-value	Test statistics	Baseline	12 weeks	P-value	Test statistics
VAS, mean (SD)	5.1 (2.2)	4.1 (2.6)	0.07	t = 1.97	4.5 (2.4)	3.7 (2.6)	0.18	t = 1.38
KOOS, mean (SD)								
Symptom	63.2 (13.4)	66.0 (13.3)	0.32	t = -1.03	69.0 (17.0)	74.6 (12.9)	0.05	t = -2.08
Pain	59.0 (16.9)	65.2 (15.7)	0.07	t = -1.91	69.0 (15.8)	76.5 (15.5)	0.02	t = -2.65
ADL	70.3 (12.7)	74.3 (15.8)	0.24	t = -1.23	77.7 (12.4)	81.5 (13.8)	0.26	t = -1.17
SP	30.6 (19.2)	42.6 (21.1)	0.01	t = -2.98	55.0 (23.9)	63.9 (24.2)	0.16	t = -1.45
QOL	39.3 (18.8)	46.0 (18.2)	0.05	t = -2.1	50.7 (18.7)	56.2 (19.1)	0.28	t = -1.11
6-meter gait								
Speed (s), mean (SD)	7.1 (2.9)	5.5 (1.3)	0.02	t = 2.83	5.4 (1.0)	5.5 (1.0)	0.68	t = -0.43
Step count, mean (SD)	12.2 (2.6)	11.3 (1.9)	0.02	t = 2.75	11.2 (1.7)	11.4 (1.4)	0.67	t = -0.43
Quadriceps torque (N), mean (SD)								
Left	23.8 (9.5)	25.0 (10.3)	0.36	t = -0.96	29.7 (9.5)	27.6 (9.7)	0.28	t = 1.13
Right	25.8 (9.2)	27.0 (10.5)	0.35	t = -0.97	29.5 (14.0)	29.8 (9.8)	0.66	t = -0.46
Quadriceps volume (cm^2^), mean (SD)								
Left	2.9 (1.0)	3.3 (0.9)	0.001	t = -4.75	3.2 (1.1)	3.4 (1.2)	0.24	t = -1.23
Right	3.0 (1.0)	3.5 (0.9)	0.001	t = -4.51	3.3 (1.3)	3.5 (1.4)	0.06	t = -2.02

In the comparisons of outcome changes between groups, there were no significant differences in VAS or PROMs, including all KOOS subscales. However, the EAA group showed significantly greater improvements in 6-meter gait speed and step count, as well as increased quadriceps muscle volume on both sides, compared to the control group (Table 3). In contrast, no significant differences were observed in quadriceps torque between the groups at 12 weeks. During the study period, no adverse events or side effects related to the intervention were observed.

**Table 3:** Comparison of changes in clinical outcomes between the EAA and control groups. Values represent changes from baseline to 12 weeks (Δ), presented as mean ± standard deviation (SD). P-values represent between-group comparisons of these changes, calculated using Student's t-tests. P-values < 0.05 were considered statistically significant. EAA, essential amino acid; VAS, visual analogue scale; SD, standard deviation; KOOS, Knee injury and Osteoarthritis Outcome Score; ADL, activities of daily living; SP, sports and recreational activities; QOL, quality of life; cm², square centimeters.

Variable	EAA	Control	Mean difference	P-value	Test statistics
VAS, mean (SD)	-1.0 (2.1)	-0.8 (2.5)	-0.21	0.78	-0.28
KOOS, mean (SD)					
Symptom	2.7 (10.9)	5.6 (11.8)	-2.91	0.45	-0.77
Pain	6.2 (13.4)	7.5 (12.3)	-1.25	0.77	-0.29
ADL	4.0 (13.4)	3.8 (14.2)	0.19	0.97	0.04
SP	12.1 (16.7)	8.9 (26.9)	3.11	0.68	0.42
QOL	6.6 (13.0)	5.6 (21.9)	1.03	0.86	0.17
6-meter gait					
Speed (s), mean (SD)	-1.5 (1.7)	0.1 (0.5)	-1.54	0.02	-2.85
Step count, mean (SD)	-0.8 (0.9)	0.1 (0.7)	-0.88	0.02	-2.53
Quadriceps torque (N), mean (SD)					
Left	1.4 (4.8)	-1.8 (5.5)	3.18	0.15	1.48
Right	2.0 (7.0)	0.6 (4.9)	1.4	0.59	0.56
Quadriceps volume (cm^2^), mean (SD)					
Left	0.8 (0.5)	0.2 (0.5)	0.59	0.01	2.87
Right	0.7 (0.5)	0.2 (0.3)	0.49	0.01	2.94

Sensitivity analyses

A complete case analysis was performed without LOCF imputation, and the results were consistent with those of the primary analysis. No significant differences were found in PROMs or quadriceps torque, whereas significant differences were maintained in quadriceps volume and step count.

Power analysis

A post-hoc power analysis was performed. For the primary outcome of VAS change, the effect size was small (Cohen's d = 0.09) with an achieved power of 0.06 at α = 0.05. For the secondary outcome of quadriceps muscle volume, the effect sizes were d = 1.19 (left) and d = 1.25 (right), yielding achieved powers of 0.79 and 0.84, respectively.

## Discussion

The most important findings of this study were that the EAA group demonstrated improved gait function and greater quadriceps muscle hypertrophy compared to the control group. However, PROMs, such as VAS and KOOS, showed no significant differences between the two groups, nor did quadriceps torque measurements.

Previous studies in perioperative settings have demonstrated the beneficial effects of EAA supplementation on muscle preservation and recovery. For example, Ueyama et al. showed that perioperative EAA supplementation (9 g/day) during TKA significantly promoted muscle volume and strength recovery compared to placebo, with effects persisting for two years [13]. Similarly, Dreyer et al. reported in a double-blind randomized controlled trial that 20 g/day of EAA reduced postoperative quadriceps atrophy following TKA [5]. These findings support the role of EAA in preventing perioperative muscle loss. Our study extends these findings by demonstrating that EAA supplementation may also promote muscle hypertrophy and improve gait function in patients receiving conservative, non-surgical treatment. Furthermore, Takeuchi et al. reported improvements in VAS and functional outcomes following oral intervention with six amino acids in patients with knee discomfort managed conservatively [10]. Consistent with this, our results showed functional benefits, including improved walking ability and increased quadriceps volume, which support the potential of EAA supplementation as part of conservative management.

The present study found no significant between-group differences in PROMs, which is consistent with previous reports showing that imaging-based improvements do not always correspond to subjective or functional benefits [5,13]. The lack of between-group differences in PROMs, despite improvements in gait function and muscle volume, suggests that the effect of EAA on subjective symptoms may be limited, or that the study was underpowered to detect such differences. Further studies with larger sample sizes or alternative dosing regimens are needed to clarify this relationship. In addition, although quadriceps torque showed a favorable trend in the EAA group, the difference was not statistically significant. In contrast, quadriceps volume demonstrated a significant between-group difference. This discrepancy may be attributable to insufficient statistical power to detect smaller effect sizes in torque.

This study has several limitations. First, as a retrospective study, selection bias is inevitable. In fact, the EAA group showed lower baseline PROMs and knee function scores (Table 2), suggesting greater initial functional impairment and pain. Although the impact may be minimal, given that our outcomes involved longitudinal changes in PROMs, this remains a significant concern. Ideally, a randomized controlled trial with comparable baseline characteristics and unbiased group allocation would be preferable. Second, as the EAA supplementation was purchased at the patients' own expense and based on their self-decision, a potential selection bias exists, as only those who could afford or were motivated to buy may have opted for supplementation. Third, there is no consensus regarding EAA dosage and timing. Previous studies have used varying EAA doses, ranging from 9 to 20 g/day, whereas our study used 8 g/day, prioritizing compliance and safety. If effects are dose-dependent, this could represent a significant limitation and may explain insufficient effects in our study. Fourth, the follow-up period was limited to three months, highlighting the need to assess medium- and long-term outcomes of sustained supplementation. Fifth, baseline nutritional status, such as serum albumin levels, was not assessed. As a result, we were unable to evaluate the potential influence of pre-existing nutritional conditions on the outcomes, which may limit the interpretation of the effect of EAA supplementation. Sixth, compliance with EAA supplementation was not systematically assessed, as intake was voluntary and self-managed in this retrospective study. This makes it difficult to assess the consistency of supplement intake and its association with treatment effects. Seventh, outcome assessors were not blinded to group allocation. As this was a retrospective study and the measurements (e.g., quadriceps torque and ultrasound imaging) were conducted as part of routine clinical practice, blinding was not implemented. This may have contributed to measurement bias. Eighth, the frequency and intensity of supervised physical therapy were not systematically documented in this retrospective study. Therefore, variability in rehabilitation exposure between participants may limit the reproducibility and interpretation of the intervention effects.

## Conclusions

Although no significant differences were observed in PROMs, EAA supplementation in the conservative treatment of knee OA demonstrated significant improvements in gait function and quadriceps muscle volume compared to the control group. These findings suggest the potential utility of EAA supplementation in the conservative management of knee OA. Further prospective studies with larger cohorts are needed to confirm these results and to establish optimal dosing strategies. Randomized controlled designs and longer follow-up periods will be crucial in addressing the limitations of the present study.
